# Sustainable Development of Hot-Pressed All-Lignocellulose Composites—Comparing Wood Fibers and Nanofibers

**DOI:** 10.3390/polym13162747

**Published:** 2021-08-16

**Authors:** Erfan Oliaei, Tom Lindström, Lars A. Berglund

**Affiliations:** 1 Wallenberg Wood Science Center, Department of Fibre and Polymer Technology, KTH Royal Institute of Technology, SE-100 44 Stockholm, Sweden; oliaei@kth.se; 2 Material and Surface Design Department, Bioeconomy and Health Division, RISE Research Institutes of Sweden, SE-114 86 Stockholm, Sweden; 3 Department of Chemistry, Stony Brook University, Stony Brook, NY 11794, USA; toml@kth.se

**Keywords:** nanocellulose, nanofibrillar/microfibrillar lignocellulose, lignin-containing wood fibers, unbleached kraft pulp, molded fiber, biocomposite, mechanical properties, cumulative energy demand (CED), sustainability

## Abstract

Low-porosity materials based on hot-pressed wood fibers or nanocellulose fibrils (no polymer matrix) represent a new concept for eco-friendly materials with interesting mechanical properties. For the replacement of fossil-based materials, physical properties of wood fiber materials need to be improved. In addition, the carbon footprint and cumulative energy required to produce the material also needs to be reduced compared with fossil-based composites, e.g., glass fiber composites. Lignin-containing fibers and nanofibers are of high yield and special interest for development of more sustainable materials technologies. The present mini-review provides a short analysis of the potential. Different extraction routes of lignin-containing wood fibers are discussed, different processing methods, and the properties of resulting fiber materials. Comparisons are made with analogous lignin-containing nanofiber materials, where mechanical properties and eco-indicators are emphasized. Higher lignin content may promote eco-friendly attributes and improve interfiber or interfibril bonding in fiber materials, for improved mechanical performance.

## 1. Introduction

Fossil-based plastics are widely used in the form of molded components, for example in appliances, cars, general industry and transport, consumer goods, packaging, and furniture [1,2]. As a society, we depend on man-made plastics although there is an increasing awareness of the need for more eco-friendly replacement materials from renewable resources. Cellulose biocomposites with thermoplastic polymer matrix are important alternatives, due to fast melt processing (e.g., injection molding) and favorable mechanical performance [3]. The polymer matrix, however, is usually petroleum-based and recycling is not always straightforward.

The concept of a fibrous material consisting only of plant fibers is therefore interesting, since the material would be completely based on renewable resources. One example within industrial materials is so-called molded pulp or molded fibers, commonly used for packaging [4]. Briefly, the technology is based on the use of a wet plant fiber “cake”, which is subjected to molding into geometrical shape in processes resembling compression molding, at ambient or elevated temperature. The traditional materials are, for instance, used in egg cartons, but recently, preparation of molded fibers of higher density has been suggested [5]. One may envision the use of hot-pressing in metal molds [6] in order to create molded plant fiber materials of low porosity and much improved mechanical properties compared with existing fiber materials.

In an early study [5], we used high cellulose content wood sulfite fibers (no lignin) and subjected the fibers to high-pressure compaction at elevated temperature to form “binder-free” all-cellulose composites, or fiber materials, without the use of any solvent. A Young’s modulus of 13 GPa and an ultimate tensile strength of 76 MPa was obtained at a porosity of around 16%, for fibers oriented random-in-the-plane. Arévalo and Peijs [7] made thicker materials from flax fibers and reported strengths approaching 90 MPa. Later holocellulose fibers from spruce were used [8], with virtually no lignin, and we obtained a modulus of 18 GPa and a tensile strength of 195MPa. An interesting question is if lignin-containing wood fibers can provide advantages compared with highly delignified wood fiber grades. One potentially beneficial effect could be that lignin has a binder function. Breakdown of lignin, e.g., cleavage of β-O-4 and β-β linkages in the course of hot-pressing, where various forms of phenols and phenoxy radicals are released, has been documented [9]. The self-bonding of lignin-derived moieties, i.e., condensation repolymerization, cross-linking and coupling to other wood components, such as hemicelluloses or cellulose possibly occurs, serving to the binder function [9]. This may improve the mechanical properties, though it may aggravate recyclability. Abe and coworkers [10] investigated hot-pressed nanocellulose films based on microfibrillated cellulose from chemo-thermomechanical wood pulp. They found that hot-pressed films showed a shiny surface, indicating that the lignin-hemicellulose mixture in the fibrils showed liquid-like flow during the molding operation.

The objective of this mini-review is to analyze the potential of lignin-containing wood fibers and nanofibers in the context of binderless hot-pressed fiber materials akin to molded fibers, but with lower porosity. Lignin-containing fibers may be more eco-friendly than high-purity cellulose wood fibers. In addition, the recent interest in nanocellulose motivates a comparison of mechanical properties and candidate applications of hot-pressed films and materials based on wood fibers or lignocellulose nanofibers.

## 2. Lignin-Containing Wood Pulp Fibers

The first fiber case considered are wood pulp fibers, which are typically 30 micrometers in diameter and 3 mm in length if they are from softwood. The kraft process is the most common process for preparation of wood fiber pulp both in Europe and in the United States [11]. Delignification of wood tissue during kraft pulping starts at the inner cell wall and in later stages it reached middle lamella and cell wall corners. This results in higher residual lignin concentration on the outer part of kraft pulp fibers [12]. Unbleached kraft pulps are less deformed than bleached pulps [13] and show comparatively fewer fiber twists, curls, and kinks [13,14,15]. In addition, unbleached kraft fibers show more limited chemical degradation compared to bleached fibers [15,16]. High-lignin unbleached kraft pulps tend not to be collapsed and are rather straight [17], may show high strength, stiffness [15], and higher carboxylic acid content (charge) [17,18]. The yield from wood chips ranges from 40% for some commercial bleached kraft pulp grades to 60% for unbleached paper and linerboard fiber grades [19]. Notice that yield is also strongly related to wood species (higher for hardwoods), chip size and quality, and pulping conditions, which might be of more importance than the degree of delignification [19]. However, the higher yield for unbleached kraft certainly results in lower cumulative energy demand [20] and also lower price than for bleached kraft wood fibers. Low use of chemicals [17], lower effluent discharge [21,22,23], and lower carbon footprint [24,25] are other advantages of unbleached kraft fibers in the context of sustainable development.

Although unbleached wood fibers are in focus, alternative wood fibers of high lignin content are interesting. Neutral sulfite semi-chemical (NSSC) pulp is mostly used for hardwoods (e.g., birch and aspen), and lignin content tends to be lower than 20% [26]. Wood chips are first chemically cooked with Na_2_SO_3_ and NaOH, and then mechanically defibrated with limited fiber damage. The yield is about 75–85% and the main product is fluting for corrugated boards.

**Figure 1:**
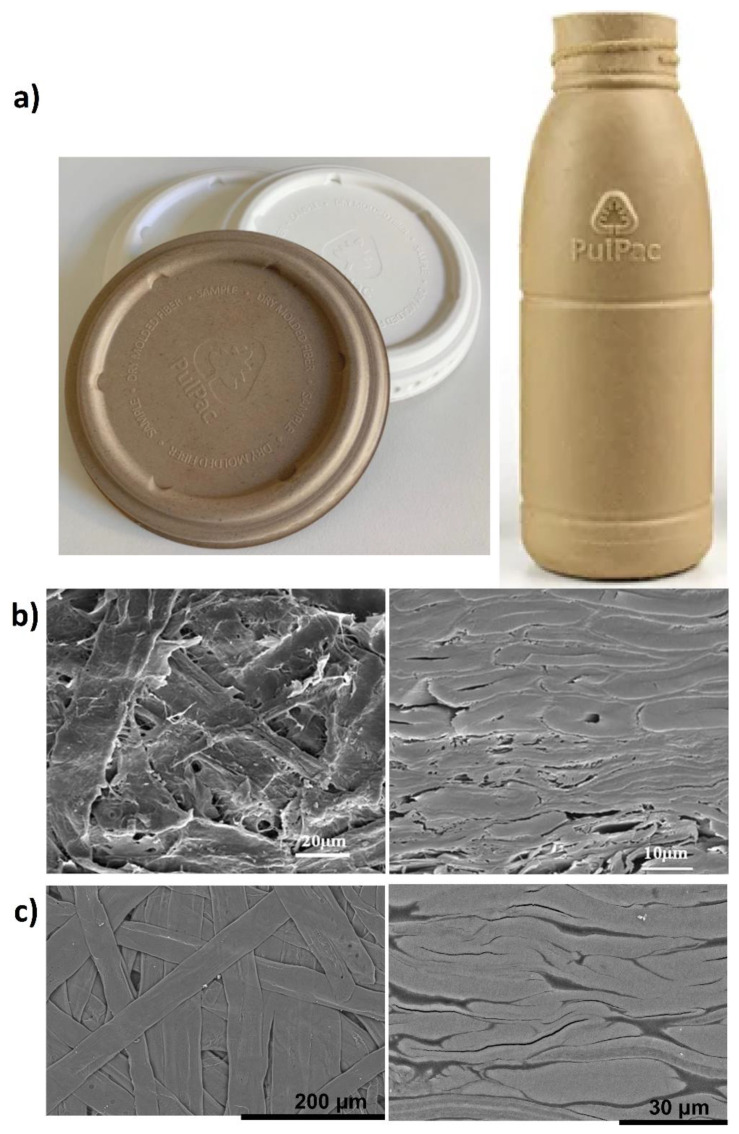
(**a**) Examples of molded pulp products in the form of lids to coffee cups and bottles [39]. Top and cross-sectional SEM images of hot-pressed molded fibers from (**b**) mildly delignified TMP fibers [32] or (**c**) unbleached kraft fibers [14]. (**a**) Reprinted with permission from PulPac AB [39] (**b**) Adapted with permission [32], Copyright 2018, Springer Nature. (**c**) Adapted with permission [14], Copyright 2021, American Chemical Society.

Chemi-thermomechanical pulp (CTMP) are wood fibers related to thermomechanical pulp (TMP), but wood chips are pretreated, usually with alkaline sodium sulfite solutions [26,27]. Chips are then steam-heated (120–170 °C) to soften the lignin and facilitate fiber separation in the disk refiner [26,28]. This results in isolation of long fibers with reduced mechanical damage, low content of fiber fragments (fines) and shives. The CTMP yield is in the range of 85–90% [28]. For application examples, bleached kraft pulp is used for quality printing and writing paper, unbleached kraft for linerboard and sack paper and CTMP for the middle ply of paperboard and boxboard [11,26]. Compared with TMP (Figure 1b), both unbleached kraft (Figure 1c) and CTMP fibers are better preserved with more limited mechanical degradation of fibers.

## 3. Molded Wood Pulp Fibers

Molded pulp is an environmentally friendly packaging material that is recyclable, compostable, and eventually biodegradable [6]. The first method for molding of wood pulp appeared in 1890 [4,29] and then later a machine to manufacture molded pulp was patented by Martin L. Keyes, from Cambridge, Massachusetts in 1903 [4,30]. Soon after, egg cartons were designed and produced by Joseph Coyle. Other early applications were related to a medical folding spoon, pastry packaging and telephone handset packaging [4,31]. Molded pulp is now formed in rather complex 3D shapes and are used for a wide range of applications, e.g., clamshells, honeycomb paperboard, end caps, trays, cups, etc. as alternatives to petroleum-based packaging products [6,32], see two examples in Figure 1a. Molded pulp bottles (containing a thin plastic lining) were recently developed [4]. The molded pulp market is predicted to have an annual growth rate of more than 4.5% during 2020–2026 [33].

Molded pulp products are categorized as (1) *thick-walled* (one smooth and one rough surface) processed via open forming mold and oven drying, e.g., for heavy item cushioning, (2) *transfer molded* (rather *thin-walled*, smooth surfaces) processed via forming mold, transferred into a take-off mold followed by oven drying, e.g., for egg trays and electronic packaging, (3) *thermoformed* (thin and dense wall, smooth surfaces) processed via partially forming the product and hot-press molding (thermoforming), e.g., for plates, bowls, dishes and specialty products such as loudspeaker membranes. Molded pulp can also be (4) *post-processed,* e.g., coated, printed, etc. [4,34]. The energy consumption and processing time of molded pulp can be considerably reduced by impulse drying, particularly for hot-pressed items [4].

Traditional molded pulp is formed by single-suction molding (thick-walled) with a typical density of 0.22–0.35 g/cm^−3^, where recycled fibers can be used [4,32]. However, high-strength molded pulp products can have densities of 0.8–1.1 g/cm^−3^ and are formed from virgin plant fibers by hot-pressing. Note the low-porosity cross-sectional morphologies of molded pulp in Figure 1b,c. High-strength molded pulp could be used for large and heavy mechanical equipment packaging, collection packaging, as well as field handling [32]. In the context of sustainable development, the use of recycled fibers extends the service life of wood fibers, which reduces carbon footprint. The cumulative energy to produce recycled fibers is 27% lower than for virgin fibers [35]. Recycling by current technologies, however, reduces quality and strength of fibers as well as the dimensional accuracy of molded products [35,36]. The shorter fibers, higher fine content, collapsed cell walls, higher degree of hornification, reduced fiber swelling, and flexibility of recycled fibers cause reduction of density, interfiber bonding, strength, and aesthetic appearance of the final product [35]. Due to quality aspects and cost, therefore, the recycling of molded fibers is typically limited to five to seven times [35,36] and is primarily used for lower quality products [4].

Literature data shows 6–17% residual lignin in the fibers may distinctly contribute to the strength, stiffness, and water resistance of molded pulp products [14,32,37]. Mild delignification of very high lignin content wood fibers (from 25% to 11%) may increase fiber diameter and reduce cell wall thickness, which facilitates densification of fibers during hot-pressing [32,38]. Although the total lignin content decreases by mild delignification, the oxygen-rich composition of the fiber outer surface reduces, i.e., the lignin content on the outer surface may actually increase. This promotes surface hydrophobicity and may promote interfiber adhesion and improve mechanical properties. Although one drawback may be the reduction of enthalpy required for thermal decomposition of the components and decreased thermal stability of the hot-pressed fiber material [32].

In the 1930s, studies of Mason showed that lignin-rich fibers subjected to heat, moisture, and pressure can fuse and create a densified hard-surface fiberboard [40]. Recent research of Joelsson et al. [26] and Oliaei et al. [14] investigated how moisturized, lignin-rich fibers can bond to form densified fiber structures. Lignin may bind fibers strongly, as Mason described, and tensile strength as a function of density can be significantly higher for hot-pressed lignin-rich fibers, particularly unbleached high-lignin kraft (as will be discussed for Figure 6b), NSSC, or CTMP pulps [14,26]. The adhesion effect of lignin in hot-pressed films is reflected in enhanced (dry and wet) strength, and water-swelling restrictions of the films [14,26].

Delignified wood fibers with essentially no lignin (holocellulose) are interesting as reference materials, although such fibers are not available industrially. Yang et al. [8] showed that hot-pressing of holocellulose fibers (high hemicellulose but low lignin content) leads to dense fiber structures (Table 1) with excellent mechanical properties. As Table 1 shows, hot-pressed fiber materials with lower porosity (higher density) show higher values of modulus and tensile strength. Strong interfiber binding leads to steeper strain-hardening and higher ultimate strength.

**Figure 2:**
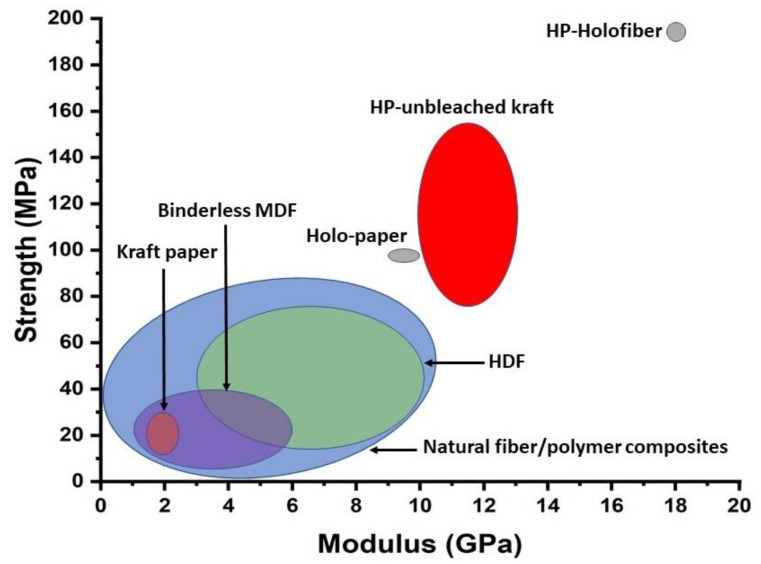
Strength vs. modulus Ashby plots for fiber materials (data for 2D-random materials from Table 1; data for natural fiber/polymer 2D and 3D-random composites from [51]).

The reasons for high mechanical properties of hot-pressed holocellulose fibers (Figure 2) include both the use of undamaged strong fibers, and strong interfiber adhesion. However, moisture/water sensitivity is a challenge for low-lignin holocellulose fibers. High-lignin unbleached kraft and NSSC fibers are often more resistant to moisture [14], are industrially available and have industrial potential for hot-pressed fiber materials. Figure 2 shows that hot-pressed “HP-unbleached kraft” fiber materials reported by the present authors [14] are considerably stronger than common randomly oriented natural fiber/polymer biocomposites, medium-density fiberboard (MDF), and high-density fiberboard (HDF). This is related to high fiber content, low porosity and the hot-press technique where no fiber damage is introduced, in contrast to melt-processed biocomposites.

## 4. Microfibrillated Lignocellulosic Nanofibers (MFLC) and Nanopaper Properties

**Figure 3:**
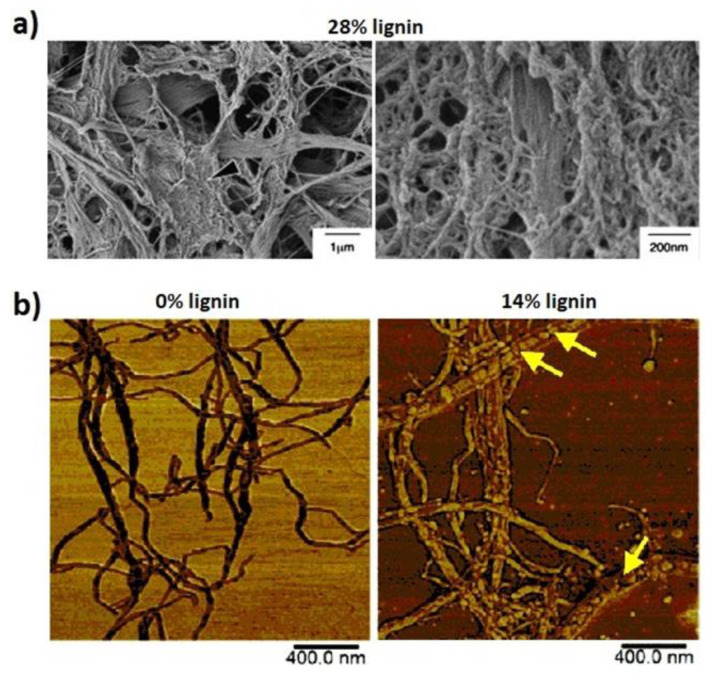
(**a**) SEM images of mechanically fibrillated CTMP (28% lignin) [10]. (**b**) AFM images of LCNF from sulfur dioxide-ethanol-water (SEW) pulp with and without lignin [57]. (**a**) Adapted with permission [10], Copyright 2009, Elsevier Ltd. (**b**) Adapted with permission [57], Copyright 2015, Royal Society of Chemistry.

In contrast to large size wood pulp fibers, microfibrillated lignocellulose nanofibers are typically only 10–50 nm in diameter and a few micrometers in length. Microfibrillated cellulose without lignin (MFC) was first produced by high-pressure homogenizing of wood fibers in the late 1970s [52]. Since then, various pretreatment methods, including enzymatic pretreatment, introduction of charged groups [53,54,55], and/or mechanical treatment [56] have been utilized to improve fibrillation. So far, very low lignin content fibers have predominantly been used for fibrillation. The pioneering study of Abe et al. [10] demonstrated fibrillation of CTMP fibers with 28% residual lignin, using one-time grinder treatment (see Figure 3a). Microfibrillated lignocellulose (MFLC, nanofibers with considerable residual lignin) of even finer diameters were later produced by mechanical treatment of sulfonated (sulfur dioxide-ethanol-water treated or SEW) pulp fibers [57] (Figure 3b), TEMPO-oxidized fibers [58], or TEMPO-oxidized and enzymatic treated fibers [59]. Note that the presence of lignin makes it more difficult to prepare well-defined nanofibrils of small diameter, as is possible for delignified wood pulp. The lignin-containing nanofibers in Figure 3 are branched and heterogeneous in diameter and shape.

**Figure 4:**
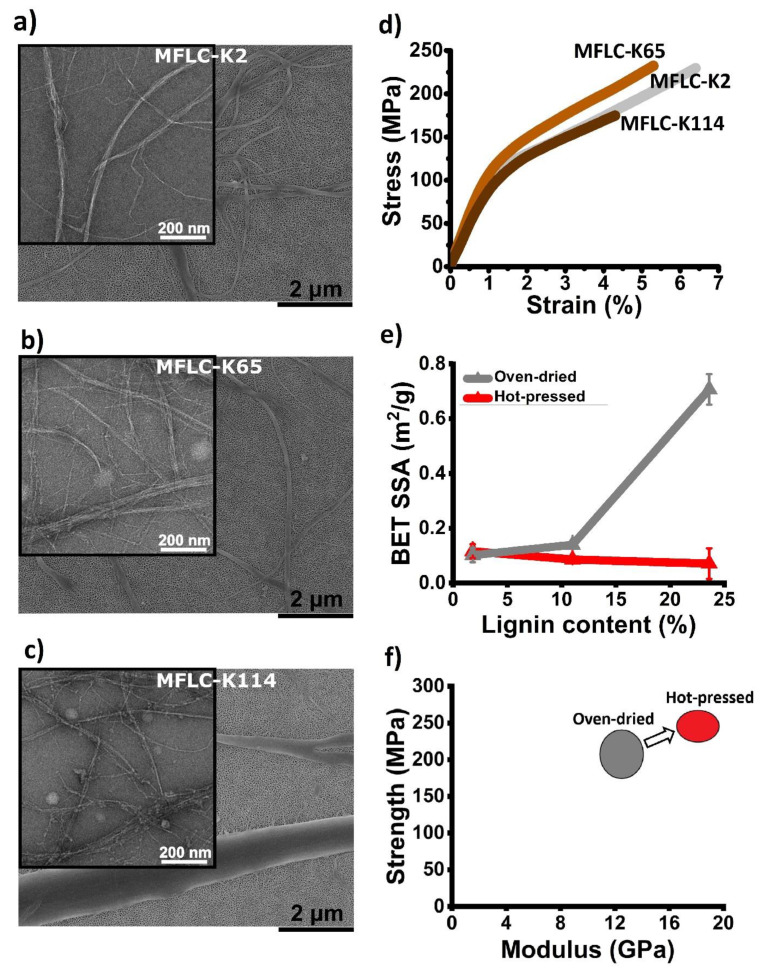
SEM and TEM images of (**a**) MFLC-K2 (2% lignin, 2.5 nm-0.8 µm fibril diameter ϕ, 42 wt% nano-sized) [14], (**b**) MFLC-K65 (11% lignin, 2.5 nm–0.8 µm ϕ, 53 wt% nano-sized) [14], and (**c**) MFLC-K114 (24% lignin, 2.5 nm–2 µm ϕ, 24 wt% nano-sized) [14]. (**d**) stress-strain curves of oven-dried (50 °C) MFLC films [17]. (**e**) BET specific surface area of the hot-pressed and oven-dried MFLC films [14]. (**f**) Mechanical properties of hot-pressed MFLC vs. oven-dried films. K2, K65, and K114 are kappa numbers, related to 2%, 11%, and 24% lignin contents, respectively. (**a**–**c**,**e**) Adapted with permission [14], Copyright 2021, American Chemical Society. (**d**) Adapted with permission [17], Copyright 2019, Springer Nature.

Previous studies on preparation of MFLC are predominantly based on either a specific starting material (e.g., wood particles or specific pulp fibers) [57,60,61,62] or specific chemical pretreatments [58,59]. Recently, we prepared MFLC from unbleached kraft pulp fibers by mechanical treatment only (see Figure 4) [17]. The process is industrially relevant due to the use of commercial pulp without any special pretreatment. The effect of residual lignin content, in the range of 2–24%, on fibrillation was carefully investigated. An optimum lignin content of 11% was found, where finer (smaller diameter) fibrils and a higher yield of nanofibrils resulted [17], combined with high lignin content compared with other lignin-containing compositions. Unbleached kraft fibers showed an almost linear relationship between lignin content and carboxyl group content (charge). The optimum lignin content for MFLC preparation is due to the balance of high charge facilitating fibrillation, and reduced lignin content, which facilitates fibrillation [17].

TEM images of MFLC with different lignin content are presented in Figure 4 [17]. Higher charge of unbleached kraft fibers resulted in generation of finer fibrils. Note that Figure 4b with kappa 65 (11% lignin) shows fine diameter MFLC fibrils, whereas the highest lignin content fibril population contains a large fraction of large cell wall fragments. Nanopaper structures prepared from the complete MFLC population, including cell wall fragments, showed high mechanical properties which were competitive with films containing only nano-sized fibrils. This suggests a strong effect from lignin as a binder so that the strain field distribution becomes fairly homogeneous also for heterogeneous MFLC populations.

As Figure 4d shows oven-dried 11% lignin-containing MFLC film (K65) showed similarly high values of tensile strength and modulus (initial slope of stress-strain curve) as bleached MFLC films (K2, 2% lignin). Hot-pressing of the moist MFLC films (compared to oven-dried films) reduced the specific surface area and porosity of the lignin-containing films, suggesting lignin flow during hot-pressing conditions (Figure 4e,f). In addition, hot-pressing may enhance lignin interfibril bonding. As a result, mechanical properties (i.e., modulus and ultimate strength) of hot-pressed MFLC films are higher than for oven-dried films. We have not been able to find other data in the literature exceeding the mechanical property data for MFLC films with 11% lignin in Figure 4d,f.

Other advantages from lignin such as reduced moisture effects, wet strength, UV absorption, reduced surface area/microporosity, and oxygen and water vapor permeability have also been shown for lignin-containing nano- and microfibrillated cellulose films [10,14,17,57,63]. These unique properties are promising for applications in packaging, water purification, binderless high-density boards and perhaps thermoformed materials.

## 5. Mechanical Property Comparison of Lignocellulosic Wood Fiber and Nanofiber Materials

**Figure 5:**
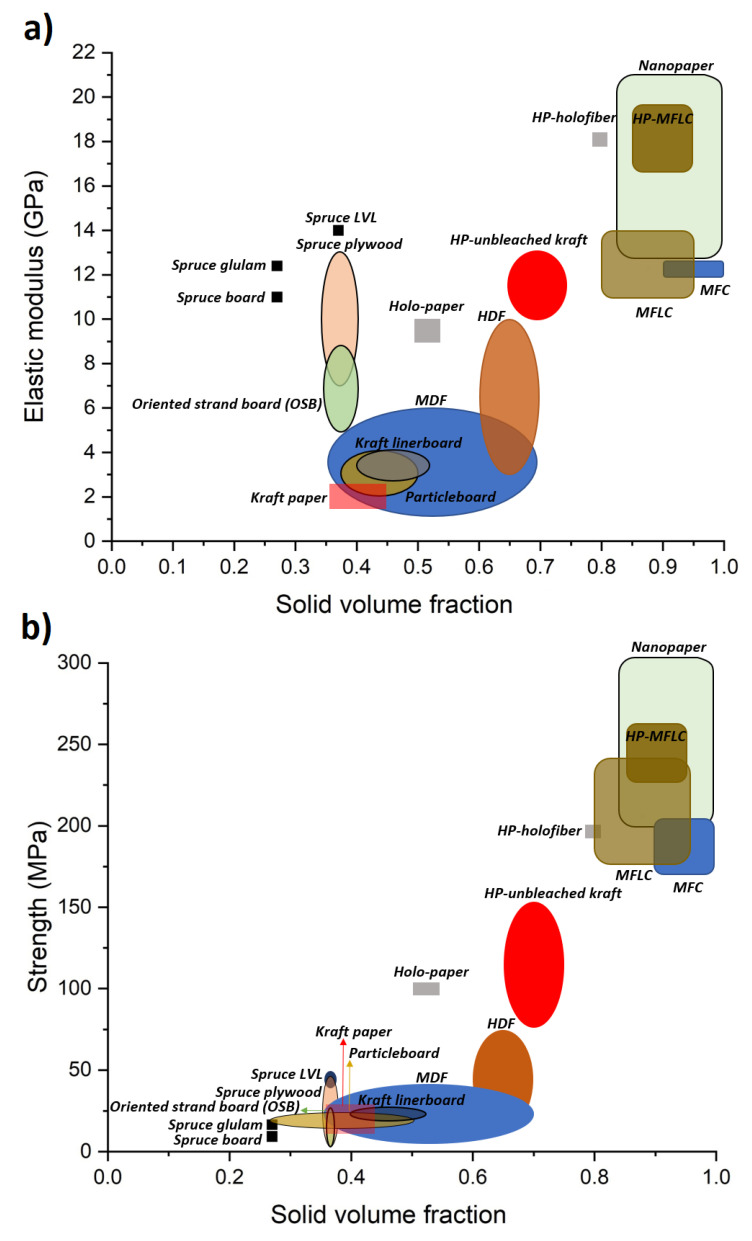
(**a**) Modulus and (**b**) strength versus solid volume fraction of different wood-based fiber materials. Data from Table 1 and refs [64,65].

It is of interest to compare mechanical properties of hot-pressed wood fiber and nanofiber materials where no binder or polymer matrix has been added. Other forest products, such as fiberboard materials, are included. Figure 5a shows modulus and Figure 5b strength versus solid volume fraction of different wood fiber materials. For modulus, one may note that laminated veneer lumber (LVL), glulam, spruce boards, and plywood are all oriented structures and modulus is measured in the fiber direction. For this reason, they show better properties for a given solid volume fraction, and they are presented for reference purposes although not strictly comparable to random-in-plane fiberboard and sheet materials. The random-in-plane materials show non-linear dependence of modulus on solid volume fraction of fibers since we expect the function to start at the origin where axes intersect. Most likely, the main reason is that fiber-fiber bonding is poor at low solid volume fractions. The red area for modulus of HP-unbleached kraft is favorably located between industrial fiberboard materials and nanofiber films. Apparently, higher fiber volume fraction would lead to improved modulus, even competitive with nanofiber films.

For strength, Figure 5b, HP-unbleached kraft is much superior to industrial materials. Again, increased fiber volume fraction (reduced porosity) of this fiber material is expected to significantly increase the tensile strength, as is apparent from the figure. It is interesting that strength of the hot-pressed MFLC films, with a significant fraction of wood cell wall fragments, is comparable with strengths of cellulosic nanofiber films with no lignin. Although the lignin component is not comparable with cellulose fibrils in terms of direct strength contribution, it possibly serves as a “resin-component” that can improve interfiber and interfibril bonding and thereby the strength of the hot-pressed fiber material as a whole.

It is difficult to directly compare properties of hot-pressed wood fiber materials with MFLC materials since the solid volume fractions are different (see Figure 5). One approach is to compare specific properties (property divided by density) but the underlying assumption of how properties scale with density is unlikely to be correct. The study reported in ref [62] has the same problem.

**Figure 6:**
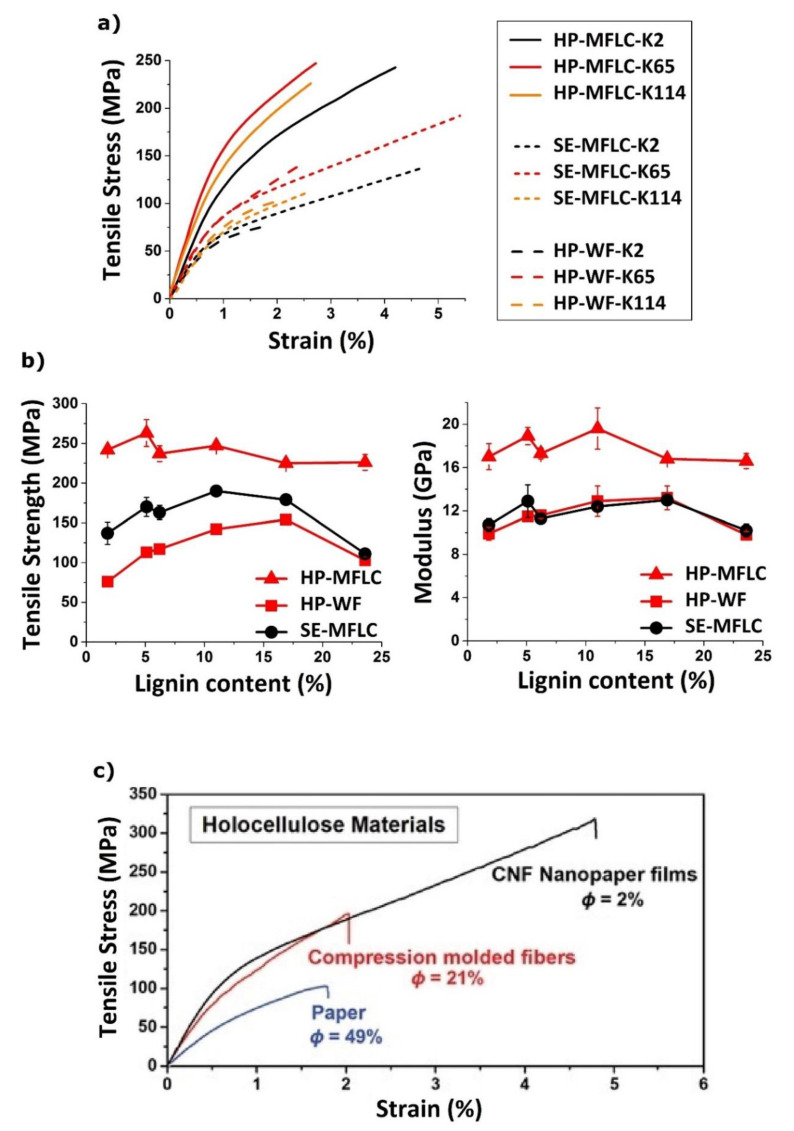
(**a**) Stress-strain curves of hot-pressed (HP)-MFLC, and comparable ≈25% porous HP-WF and solvent-exchanged (SE)-MFLC films with different kappa numbers/lignin contents from unbleached kraft pulp [14]. (**b**) Ultimate tensile strength and modulus of HP-MFLC, HP-WF, and SE-MFLC films [14]. (**c**) Stress-strain curves of holocellulose fiber materials (with almost no lignin): high porosity (ϕ ≈ 49%) fiber (Paper), low porosity (ϕ ≈ 21%) compression-molded fibers, and very low porosity (ϕ ≈ 2%) CNF Nanopaper films [66]. (**a**,**b**) Adapted with permission [14], Copyright 2021, American Chemical Society. (**c**) Adapted with permission [66], Copyright 2020, Wiley-VCH Verlag GmbH & Co.

Recently, we made hot-pressed wood fiber materials and then prepared nanofiber materials using solvent exchange and drying from acetone to obtain the same overall porosity (25%) and solid volume fraction as for the fiber materials, to make direct comparison possible. In Figure 6a, three fiber materials are compared, and they all have three different lignin contents (9 materials). The three fiber materials are hot-pressed HP-MFLC films (5–13% porosity), hot-pressed wood fibers (HP-WF) of about 25% porosity, and solvent exchanged (SE-MFLC) films of similar porosity. The hot-pressed low porosity HP-MFLC nanocellulose films are the strongest (≈250 MPa) with the highest modulus (very high, ≈20 GPa). In Figure 6a, intermediate lignin content (11%) provides the best mechanical properties, where fine fibril diameter is perhaps combined with good lignin binder function.

It is interesting to note that SE-MFLC nanocellulose films have similar modulus as HP-WF wood fiber materials of similar porosity. This confirms that nanocellulose films usually do not show advantages to comparable wood fiber materials in terms of modulus. The SE-MFLC films in Figure 6a show higher strain to failure than HP-WF because of smaller defect/pore size and also higher ultimate strength because of the strain-hardening behavior in the stress-strain curve. Again, the K65 composition with 11% lignin shows the best properties for both materials in Figure 6a. The key observation is still that HP-WF shows excellent properties and may be a class of materials more suitable than MFLC to molded products.

The effect of residual lignin content on mechanical properties of MFLC and WF films is of interest (see Figure 6b). For hot-pressed MFLC nanocellulose films, the effect is not strong, which indicates positive “binder” effects. Data also show that high lignin content films may approach the mechanical properties of cellulosic films, with added effects from the lignin binder, e.g., on maintaining film integrity under wet conditions [14]. Furthermore, these films are based on coarse MFLC fibrils, yet properties in Figure 6 are approaching those of cellulose nanofibril (CNF) films without lignin, based on finer diameter fibrils. For the SE-MFLC nanocellulose and HP-WF wood fiber materials in Figure 6b of about 25% porosity, it is interesting that ultimate strength and modulus may improve with lignin content up to about 17% lignin. At the highest lignin content of 24%, the materials showed substantial inhomogeneity with larger voids and local thickness variations. The potentially positive effects from lignin are encouraging in the context of sustainable development since lignin-containing fibers and nanofibers show higher yield (remaining weight fraction of the original wood tissue) and require less chemicals than wood fibers from bleached kraft.

Figure 6c shows related data illustrating the effect of porosity on the mechanical properties of analogous holocellulose fiber materials [66], namely “paper”, compression-molded fibers and CNF films. Although these materials contain no lignin, the main message also for lignocellulosics is that wood pulp fiber materials have significant mechanical property potential, provided the porosity could be reduced.

## 6. Aspects of Sustainable Development

Sustainable development considerations are important in new materials research and development efforts. It is important to select materials based on parameters related to environmental impact. Following Ashby [67], we use two simple metrics of environmental stress: cumulative energy demand (CED) to make a material (embodied energy in Ashby’s terminology) and global warming potential (GWP) (carbon footprint is the parameter used by Ashby). We also add water depletion (WD) since this is important for lignocellulosic materials. To simplify the scope, we estimate effects from replacement of fossil-based materials such as plastics and glass fiber-reinforced composites by molded fibers and nanocellulose. The mechanical properties presented in the present study for molded fibers and nanocellulose films are certainly comparable even to glass fiber reinforced plastics [68]. For comparable chopped strand mat/thermoset composites and sheet molding compounds with fibers oriented random in-plane, the typical modulus is in the range 5–13 GPa, and the typical tensile strength is 50–150 MPa. Mechanical properties are also significant for sustainability of semistructural materials, e.g., in automotive applications since better properties mean that the product can have reduced thickness, which makes the car lighter and reduces fuel consumption.

More in detail, GWP is a measure of accumulated absorbed heat over time (e.g., 100 years), following the emission of greenhouse gases (e.g., related to material production) [69]. Here, it is expressed as CO_2_ equivalent (CO_2-_eq), so that other greenhouse gases than CO_2_ can be considered. WD is a measure of water consumed during materials processing. “Cradle-to-gate” life cycle analysis of CNF/MFC, including extraction of the raw materials, the use of chemicals, and the processes, has been reported in several studies [70,71,72,73,74]. Since preparation of MFC or CNF nanocelluloses has wood pulp fibers as the starting materials, the environmental impact of CNF/MFC is obviously much higher. CED, GWP, and WD eco-indicators related to the production of wood fibers are much lower (see Figure S1). Foroughi et al. [73] suggested that reduction of chemical and solvent usage in modified treatments could reduce eco-indicators of nanocellulose. Because of higher cost, industrial nanocellulose utilization of CNF or MFC in materials is primarily for barrier films, coatings, adhesives, and additives in paper/board, high-tech materials and devices [75]; paints and various gels are also examples of applications in materials. The challenge of unfavorable eco-indicators for nanocelluloses also needs to be addressed in order to improve the prospects for nanocelluloses.

Unlike most previous studies, we also estimated the cumulative energy demand of CNF/MFC at an industrial scale (the energy needed to make the material). Although we used data from the studies referenced in the previous section, our critical assumptions are different: negligible material (yield) loss and 80% recycling of solvents. This resulted in much lower values. Table 2 reports cumulative energy demand of MFC and CNF produced at “industrial scale”. In the first column, the CED for bleached low-lignin kraft pulp fibers in dry state is reported as 14 MJ/kg for modern pulp mills, and about 30% lower for unbleached kraft fibers, 9 MJ/kg. These are low and very favorable values. The unbleached pulp CED may be conservative since the lignin content is low for the selected case. One may note that pulp mills using old technology may require up to three times the listed energies, and for comparison, glass fiber/thermoset composites typically have a CED of ≈110 MJ/kg [76]. The composite molding energy is around 10 MJ/kg [76], and molded wood fibers can probably be processed using similar or even lower energies. The nanocelluloses in Table 2, MFC and CNF require higher CEDs: 105 MJ/kg for mechanically disintegrated bleached pulp and 28.8 MJ/kg for TEMPO-oxidized CNF. Although the CNF is easier to disintegrate due to the oxidation, the chemicals used are increasing the CED. The data for nanocelluloses in Table 2 is much lower than most previous estimates, due to the assumptions made for industrial production. Still, the data provide arguments to further investigations of molded wood fiber materials for semi-structural applications. The MFLC reported in the bottom row of Table 2 is very interesting. Although physical properties are very good for MFLC and competitive with other nanocelluloses, the high energy defibrillation problem needs to be solved.

Another route to reduce environmental stress is to design recyclable materials. We have shown that low lignin holocellulose fiber materials can be readily recycled, since interfiber and interfibril bonding are dominated by hemicelluloses, which are swelling in water [45]. For lignin-containing fibers and fibrils, this is more difficult. In fact, it was not possible to recycle MFLC nanopaper films [14]; most likely the lignin binder function prevented this. Hot-pressed wood fibers from unbleached pulp with 17% lignin were recycled three times [14], although strength was decreased due to fiber damage. It seems that the lignin binder function was still in operation and made recycling possible although fibers were shortened and damaged.

## 7. Conclusions and Perspective

Lignin-containing wood fibers and nanofibers have not received the attention given to low lignin bleached pulp fibers and related nanofibers. Compression-molded wood fibers (hot-pressed) without added polymer binder not only show high modulus (≈13 GPa) and tensile strength (≈150 MPa), but also very low cumulative energy demand (≈9 MJ/kg). The additional molding energy for the material is roughly of the same magnitude (≈10 MJ/kg). The lignin component promotes interfiber bonding and reduces moisture sensitivity. Compared with glass fiber/thermoset composites (e.g., sheet molding compounds, SMC), the density is lower (1130 kg/m^3^ compared with 1600–1900 kg/m^3^), modulus is similar (≈13 GPa) but strength higher (150 compared with 100 MPa). Molded SMC products have a CED of ≈125 MJ/kg, and corresponding values for molded unbleached pulp will be less than 20% of those for SMC. Furthermore, molded fibers are biodegradable and can be recycled, which is not possible for thermoset composites. It would be interesting to further investigate molded fibers from annual plants, since such fibers can have better mechanical properties than wood fibers.

Mechanically defibrillated MFLC nanofibers can be made into hot-pressed films with a modulus of ≈20 GPa and a strength of ≈250 MPa. The lignin binder function and the ability for liquid flow appear to compensate for heterogeneous nanofiber size distribution. Recycling appears to be difficult for MFLC nanopaper films, without severe downgrading. The MFLC defibrillation energy from unbleached pulp is also excessively high, which means that the MFLC production problem is not solved. TEMPO-oxidation is one route towards addressing this challenge [58].

For future efforts on compression-molded wood fibers and nanofibers containing lignin, parameters quantifying environmental stress should be analyzed. The binder function of lignin makes it possible to improve moisture durability and possibly service life, compared with low lignin fiber materials. The main microstructure advantage of hot-pressed MFLC compared with lignocellulose wood fibers is lower porosity. The small size of MFLC nanofibers furthermore reduces defect size so that the tensile strength can reach 250 MPa. From the point of view of environmental stress, however, compression-molded wood fibers are much superior to MFLC materials and deserve further investigation. MFLC may be more suitable in coatings, in films, as an adhesive component and in high-technology applications, where the added performance may justify cost, and environmental stress aspects may be less important due to the small amount of material used.

## Figures and Tables

**Table 1:** Young’s modulus, ultimate strength, porosity, and elongation at break of 2D-random fiber materials.

Sample	Young’s Modulus (GPa)	Ultimate Strength (MPa)	Porosity (%)	Elongation at Break (%)	Ref
Binder-less MDF	1–6	5–40	30–65	-	[41]
Kraft paper	~2	12–30	55–65	2–4	[42,43,44,45]
Holo-paper	9–10	~100	45–50	1.5–2	[45,46]
Hot-pressed (HP)-NSSC	-	55–85	40–45	2–2.5	[26]
HDF	3–10	15–75	30–40	<1	[47,48,49,50]
HP-unbleached kraft	-	75–95	~35	3–4	[26]
HP-unbleached kraft	10–13	110–155	~25	2–2.5	[14]
HP-Holo-fiber	18	195	~20	2	[8]

**Table 2:** Cumulative energy demand of wood fibers and MFC/CNF at “industrial scale” fabrication (see supporting information).

	Pulp Fiber CED (MJ/Kg)	Chemicals/Water Energy (MJ/Kg)	Fibrillation Energy (MJ/Kg)	Mix/Wash Energy (MJ/Kg)	Total CED (MJ/Kg)
Bleached kraft pulp (BKP)	14	-	-	-	14
MFC from BKP pulp	14	1	90	-	105
TEMPO-CNF from BKP pulp	14	7.1	5.4	2.3	28.8
Unbleached kraft pulp (UBKP)	9	-	-	-	9
MFLC from UBKP pulp	9	-	>>90 *	-	>>99 *

* CED = cumulative energy demand; Fibrillation by high-pressure homogenization; * In ref [17], homogenization was carried out at low concentration, so that fibrillation energy becomes excessively high.

## Data Availability

The data presented in this study are available on request from the corresponding author.

## Supplementary Materials

The following are available online at https://www.mdpi.com/article/10.3390/polym13162747/s1, Figure S1: Comparison of eco-indicators of bleached kraft wood fiber, and common CNF/MFC (with enzymatic and carboxymethylation pretreatments, or without pretreatment), Figure S2: Evolution of weight fraction of nanocellulose yield for various pretreatments of the wood fibers used, Figure S3: Cradle-to-gate LCA system boundary of nanocellulose colloidal dispersion production, Table S1: Summary of the estimated fibrillation energy of MFC/CNF at industrial scale production, Table S2: Energy use for the no-pretreatment route, Table S3: Energy use for the enzymatic route, Table S4: Energy use for the TEMPO-oxidation route, and Table S5: Energy use for the carboxymethylation route.
