# Supplementary material for: Sustainable Development of Hot-Pressed All-Lignocellulose Composites—Comparing Wood Fibers and Nanofibers
Source: Polymers (Basel). 2021 Aug 16;13(16):2747. doi: 10.3390/polym13162747 (PMC8399967; doi:10.3390/polym13162747)
Supplement: Supplementary file 1 [file polymers-13-02747-s001.zip › polymers-1331178-supplementary.pdf]

Supplementary Materials

Sustainable Development of Hot-Pressed All-Lignocellulose Composites—Comparing Wood Fibers and Nanofibers

Erfan Oliaei [1,2], Tom Lindström [3] and Lars A. Berglund [1,*]

[1] Wallenberg Wood Science Center, Department of Fibre and Polymer Technology, KTH Royal Institute of Technology, SE-100 44 Stockholm, Sweden; oliaei@kth.se
[2] Material and Surface Design Department, Bioeconomy and Health Division, RISE Research Institutes of Sweden, SE-114 86 Stockholm, Sweden
[3] Department of Chemistry, Stony Brook University, Stony Brook, NY 11794-3400, USA; toml@kth.se
* Correspondence: blund@kth.se; Tel.: +46-8-790-6000

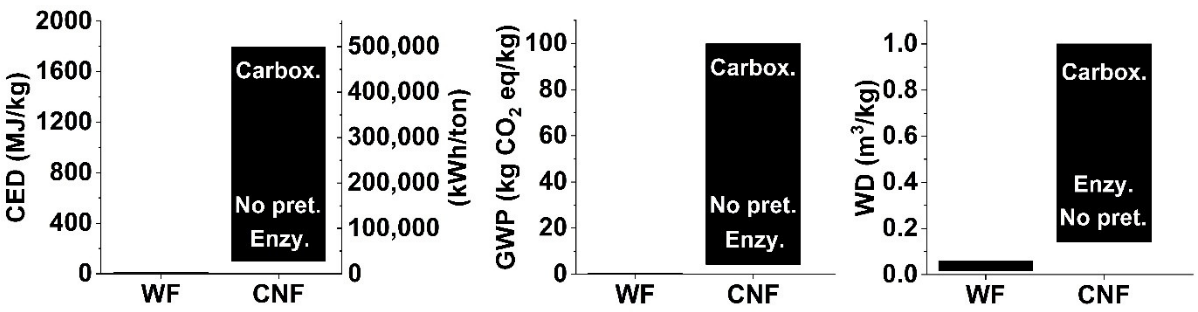

Figure S1. Comparison of eco-indicators of bleached kraft wood fiber, and common CNF/MFC (with enzymatic and carboxymethylation pretreatments, or without pretreatment) from references [1–5]; CED = cumulative energy demand, GWP = global warming potential, WD = water depletion. The denominator in the units is weight of final material. Adapted with permission [1], Copyright 2021, American Chemical Society.

Cumulative Energy Demand for Industrial Production of MFC or CNF from Different Treatment Routes

Life cycle assessment (LCA) of cellulose nanowhiskers by de Figueirêdo et al. [6] estimates 1,800–16,000 MJ/Kg cumulative energy demand. Li et al. [5] calculated cumulative energy demand of 3,500–17,600 MJ/Kg (1,000–1,700 MJ/Kg for industrial-scale production) for CNF/MFC fabrication. Nguyen [7] and Arvidsson et al. [3] reported cumulative energy demand of 90–2,100 MJ/Kg for CNF/MFC fabrication. Although these absolute values are high, they are still low compared to most other nanomaterials [7].

A lot of the information from the above-mentioned studies was used in our estimates. However, our critical assumptions for industrial-scale fabrication resulted in considerably lower values. The factors which made major differences are assuming no material (yield) loss and 80 % recycling of solvents. It should also be noted that in our calculations, we only consider cumulative energy demand of fabrication and not the quality of the prepared MFC/CNF, i.e. dimensions, chemical compositions, etc. For instance, Fig. S2 shows the weight fraction of nanocellulose content in the produced MFC/CNF gel depends on energy input. The rest of the material is typically wood fiber fragments which could be of microscale. It is apparent that chemical pretreatment facilitates so that the final yield of fine fibrils becomes high, whereas enzymatically pretreated fibers result in coarser MFC fractions. However, here we have considered an average energy demand, disregarding the yield of fine nanocellulose fibrils.

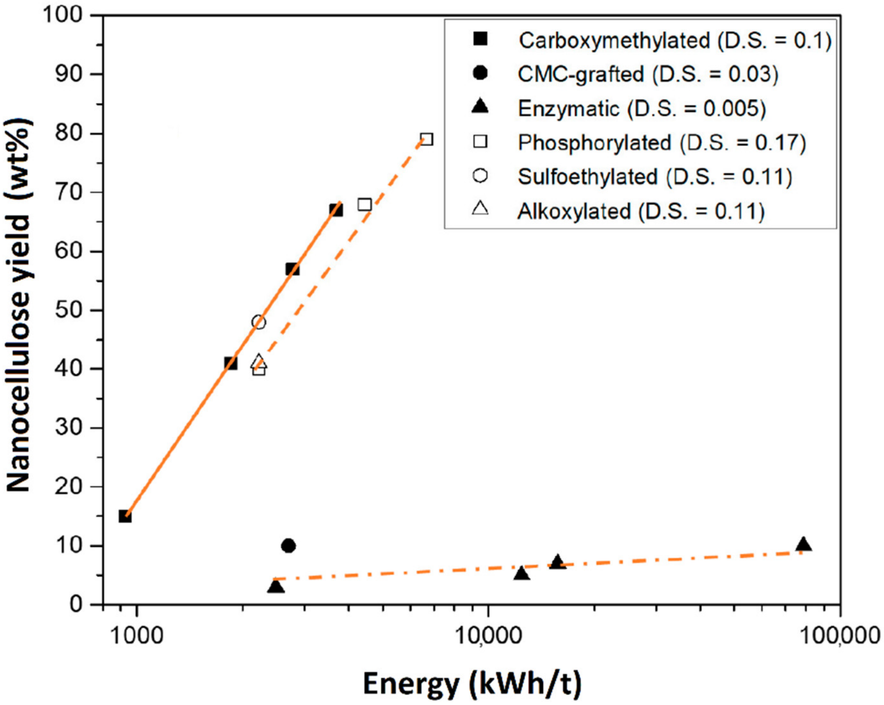

Figure S2. Evolution of weight fraction of nanocellulose yield for various pretreatments of the wood fibers used. Figure from [8].

"Cradle-to-gate" boundaries are commonly used for life cycle assessment of materials like nanocellulose, which have a wide variety of applications. This boundary includes energy demand for the extraction of the raw materials, chemicals, and various processing and drying steps, but not the use or disposal phase. This boundary is shown in Fig. S3, representing the steps related to the nanocellulose fabrication, starting from wood fiber, followed by chemical modification, washing, and mechanical fibrillation until the nanocellulose water dispersion is ready to deliver at the "factory gate" [5].

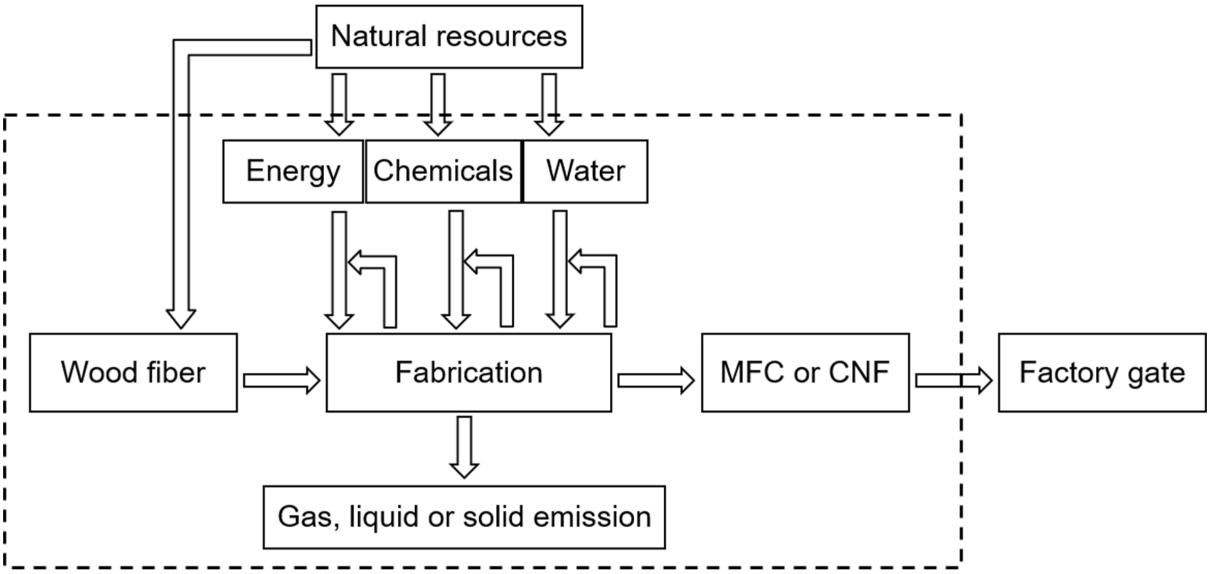

Figure S3. Cradle-to-gate LCA system boundary of nanocellulose colloidal dispersion production.

Main assumptions for cumulative energy demand assessment:

1- Transport energy of raw materials and chemicals is negligible to total cumulative energy demand. This agrees with studies of Nguyen [7] and Arvidsson et al. [3]. Their studies show that transportation energy demand at different scenarios is mostly below 3 % of total cumulative energy demand.
2- The energy demand of each raw material or chemical is considered the average of different extraction/preparation routes. For instance, energy demand associated with wood fibers considered an average sulfite or kraft pulp in modern mills [2,9,10]; and for ethanol, the average from ethylene or corn preparation routes was considered.
3- According to references [5,11], for the industrial scenario, an 80 % solvent recycling relative to lab experiments should be considered.
4- Yield loss was considered negligible. Isogai et al. [12] reported a yield of > 90 % for TEMPO-oxidized CNF in the lab experiment. No weight loss was considered for the LCA study of enzymatic and no-pretreated CNFs [13]. Li et al. [5] estimated a total weight loss of below 3 % for CNF production.
5- Homogenization energy was considered the relevant industrial fibrillation method of pretreated fibers and energy demand of auxiliary mechanical treatments (e.g. refining, beating,…) was considered insignificant or included in the homogenization energy. For instance, in Nguyen's study [7], the energy demand for pre-refining of chemically or enzymatically treated fibers is very small compared to homogenization. The study of Li et al. [5] showed that energy demand of sonication is much larger than homogenization, and as homogenization has already industrial use, it is considered the suitable industrial approach for CNF production.

Fibrillation Energy demand:

For calculation of homogenization energy demand, we have used the previous studies and made the following assumptions:

For the homogenization of non-pretreated fibers, energy consumption of 2300–70,000 KWh/ton was reported [14–19], although the range of 20,000–30,000 KWh/ton is more common [17]. Fibrillation of non-pretreated fibers using a micro-grinder is more energy-efficient, though the quality might differ. Eriksen et al. [15] and Spence et al. [18] calculated energy consumption of 1500–24,000 KWh/ton using

a micro-grinder instead of a homogenizer. Due to the comparison aspect, we considered the homogenization route and the average value of 25,000 KWh/ton (90 MJ/Kg) for fibrillation of non-pretreated fibers.

For carboxymethylated fibers, one pass of homogenization at a concentration of 2–8 wt % reported to have an energy demand of 500–2300 KWh/ton [14,16]. For two passes of homogenization at a concentration of 0.5 wt %, a much larger value was reported [5]. Hence, we considered 2300 KWh/ton as homogenization energy demand of carboxymethylated fibers.

For the enzymatically treated fibers, homogenization energy demand of 1500–11,000 KWh/ton was reported [13,16]. Hence, we considered 6000 KWh/ton as the average homogenization energy of enzymatic CNF.

For TEMPO-oxidized fibers, depending on the type of homogenizer and concentration of the suspensions, energy demand was reported in the range of 500–4900 KWh/ton [13,14]. It is well-known that TEMPO-oxidized fibers are very easy to homogenize, especially when they are highly charged. Isogai et al. [12] reported energy demand of < 1950 KWh/ton (7 MJ/Kg). Hence, we considered an average number toward the low side of this range, i.e., 1500 KWh/ton.

Table S1. Summary of the estimated fibrillation energy of MFC/CNF at industrial-scale production.

MFC/CNF Type	Fibrillation Method	Energy Consumption (KWh/ton)	Energy Consumption (MJ/Kg)
**No-pretreated**	High-pressure homogenization	25,000	90
**Enzymatic**	High-pressure homogenization	6000	21.6
**Carboxymethylated**	High-pressure homogenization	2300	8.3
**TEMPO-oxidized**	High-pressure homogenization	1500	5.4

Cumulative energy demand:

Further data used for the calculations in the tables below was extracted from studies and references [3,5,7,13,20–25].

Table S2. Energy use for the no-pretreatment route.

	Energy Use (MJ/kg)	Mass (kg/kg CNF)	Energy Use (MJ/kg CNF)
**Input materials**			
Wood fiber	14	1	14
Deionized water	0.01	100	1
			**15**
**Manufacturing processes**			
Homogenization			90
			**90**
**Total**			
			**105**

Table S3. Energy use for the enzymatic route.

	Energy Use (MJ/kg)	Mass (kg/kg CNF)	Energy Use (MJ/kg CNF)
**Input materials**			
Wood fiber	14	1	14
Deionized water	0.01	133	1.33
Enzyme	120	0.00017	0.02
Trisodium phosphate	21.4	0.042	0.9
			**16.25**
**Manufacturing processes**			
Enzyme incubation & denaturation			11
Homogenization			21.6
			**32.6**
**Total**			
			**48.85**

Table S4. Energy use for the TEMPO-oxidation route.

	Energy Use (MJ/kg)	Mass (kg/kg CNF)	Energy Use (MJ/kg CNF)
**Input materials**			
Wood fiber	14	1	14
TEMPO	Not available	0.0014-0.016	-
Sodium hypochlorite	10	0.42	4.2
Sodium bromide	0.58*	0.08	0.05
Sodium hydroxide	18.7	0.079	1.47
Deionized water	0.01	137	1.37
Hydrochloric acid	17.3	0.05*	
			**21.09**
**Manufacturing processes**			
Mixing, washing			2.3
Homogenization/agitation			5.4
			**7.7**
**Total**			
			**28.79**

*Due to lack of data, energy use of NaBr was considered as NaCl.

Table S5. Energy use for the carboxymethylation route.

	Energy Use (MJ/kg)	Mass (kg/kg CNF)	Energy Use (MJ/kg CNF)
**Input materials**			
Wood fiber	14	1	14
Deionized water	0.01	476 (× 20 %)*	4.76 (× 20 %)
Ethanol	55	28.7 (× 20 %)*	1578.5 (× 20 %)
Monochloroacetic acid	44	0.09	3.96
Isopropanol	32.7	17.9 (×20 %)*	585.33 (× 20 %)
Sodium hydroxide	18.7	0.15	2.81
Methanol	33.2	3.6 (× 20 %)*	119.52 (× 20 %)

Acetic acid	43.1	0.11	4.74
Sodium carbonate	13.8	0.73	10.07
			**493.2**
**Manufacturing processes**			
Heat, mixing, washing			2.3
Homogenization			8.3
			**10.5**
**Total**			
			**503.7**

* 80 % solvent recycling.

As shown in other studies, the difference in cumulative energy demand of wood fibers and MFC/CNF at lab scale fabrication is very large. However, the cumulative energy demand estimation of MFC/CNF produced at industrial-scale shows some CNF/MFC grades could possibly be produced at 2–3 times the energy demand for wood fibers. One should note that the final nanocellulose product is a water dispersion, and drying will require perhaps an additional 25 % energy compared with the total CED. This is significantly lower than the energy demand for most other nanomaterials.

References

1. Oliaei, E.; Berthold, F.; Berglund, L.A.; Lindström, T. Eco-friendly high-strength composites based on hot-pressed lignocellulose microfibrils or fibers. *Acs Sustain. Chem. Eng.* **2021**, *9*, 1899–1910, doi:10.1021/acssuschemeng.0c08498.
2. Brundage, A.; Evans, C.; Lizas, D.; Freed, R. OECD global forum on environment: Sustainable materials management; Organisation for Economic Co-operation and Development (OECD): Mechelen, Belgium, **2010**. https://www.oecd.org/env/waste/49804908.pdf (accessed 20 October, 2020).
3. Arvidsson, R.; Nguyen, D.; Svanström, M. Life cycle assessment of cellulose nanofibrils production by mechanical treatment and two different pretreatment processes. *Environmental Science & Technology* **2015**, *49*, 6881–6890, doi:10.1021/acs.est.5b00888.
4. Suhr, M.; Klein, G.; Kourti, I.; Gonzalo, M.R.; Santonja, G.G.; Roudier, S.; Sancho, L.D. Best available techniques (BAT) reference document for the production of pulp, paper and board; Institute for Prospective Technological Studies: Luxembourg, **2015**. https://eippcb.jrc.ec.europa.eu/sites/default/files/2019-11/PP_revised_BREF_2015.pdf (accessed 20 October, 2020).
5. Li, Q.; McGinnis, S.; Sydnor, C.; Wong, A.; Renneckar, S. Nanocellulose life cycle assessment. *Acs Sustain. Chem. Eng.* **2013**, *1*, 919–928, doi:10.1021/sc4000225.
6. de Figueirêdo, M.C.B.; Rosa, M.d.F.; Ugaya, C.M.L.; Souza Filho, M.d.S.M.d.; Silva Braid, A.C.C.d.; Melo, L.F.L.d. Life cycle assessment of cellulose nanowhiskers. *J. Clean. Prod.* **2012**, *35*, 130–139, doi:10.1016/j.jclepro.2012.05.033.
7. Nguyen, T.D. Life cycle energy assessment of wood-based nano fibrillated cellulose. Master thesis, Chalmers University of Technology, Gothenburg, Sweden, **2014**, ESA report No. 2014:5. https://hdl.handle.net/20.500.12380/200195.
8. Lindström, T. A proposition for the estimation of the maximum tensile strength of variously charged nanocellulosic film materials provided by vacuum filtration. *Nanomaterials* **2021**, *11*, 543, doi:10.3390/nano11020543.
9. Life Cycle Assessment of Deinked and Virgin Pulp; ENVIRON International Corporation: Denver, USA. https://www.greenamerica.org/sites/default/files/2018-03/NatGeo-LCA-Report-2013.pdf.
10. Benchmarking energy use in canadian pulp and paper mills; Natural Resources Canada: Ottawa, Canada, **2012**. https://www.nrcan.gc.ca/sites/www.nrcan.gc.ca/files/oee/pdf/industrial/technical-info/benchmarking/pulp-paper/pdf/benchmark-pulp-paper-e.pdf.
11. Piccinno, F.; Hischier, R.; Seeger, S.; Som, C. From laboratory to industrial scale: A scale-up framework for chemical processes in life cycle assessment studies. *J. Clean. Prod.* **2016**, *135*, 1085–1097, doi:10.1016/j.jclepro.2016.06.164.

12. Isogai, A.; Saito, T.; Fukuzumi, H. TEMPO-oxidized cellulose nanofibers. *Nanoscale* **2011**, *3*, 71–85, doi:10.1039/c0nr00583e.
13. Hohenthal, C.; Ovaskainen, M.; Bussini, D.; Sadocco, P.; Pajula, T.; Lehtinen, H.; Kautto, J.; Salmenkivi, K. *Final assessment of new nano-enhanced products*; VTT Technical Research Center of Finland: Finland, 2012.
14. Ankerfors, M. Microfibrillated cellulose: Energy-efficient preparation techniques and key properties. Licentiate thesis, KTH Royal Institute of Technology, Stockholm, Sweden, 2012, ID: diva2:557668. https://www.diva-portal.org/smash/get/diva2:557668/FULLTEXT01.pdf.
15. Eriksen, Ø.; Syverud, K.; Gregersen, Ø. The use of microfibrillated cellulose produced from kraft pulp as strength enhancer in TMP paper. *Nord. Pulp Pap. Res. J.* **2008**, *23*, 299–304, doi:10.3183/npprj-2008-23-03-p299-304.
16. Klemm, D.; Kramer, F.; Moritz, S.; Lindström, T.; Ankerfors, M.; Gray, D.; Dorris, A. Nanocelluloses: A new family of nature-based materials. *Angew. Chem. Int. Ed.* **2011**, *50*, 5438–5466.
17. Siró, I.; Plackett, D. Microfibrillated cellulose and new nanocomposite materials: A review. *Cellulose* **2010**, *17*, 459–494, doi:10.1007/s10570-010-9405-y.
18. Spence, K.L.; Venditti, R.A.; Rojas, O.J.; Habibi, Y.; Pawlak, J.J. A comparative study of energy consumption and physical properties of microfibrillated cellulose produced by different processing methods. *Cellulose* **2011**, *18*, 1097–1111, doi:10.1007/s10570-011-9533-z.
19. Turbak, A.F.; Snyder, F.W.; Sandberg, K.R. Microfibrillated cellulose, a new cellulose product: Properties, uses and commercial potential. *J. Appl. Polym. Sci. Appl. Polym. Symp.* **1983**, *37*, 815–827.
20. Saito, T.; Kimura, S.; Nishiyama, Y.; Isogai, A. Cellulose nanofibers prepared by TEMPO-mediated oxidation of native cellulose. *Biomacromolecules* **2007**, *8*, 2485–2491, doi:10.1021/bm0703970.
21. Wågberg, L.; Decher, G.; Norgren, M.; Lindström, T.; Ankerfors, M.; Axnäs, K. The build-up of polyelectrolyte multilayers of microfibrillated cellulose and cationic polyelectrolytes. *Langmuir* **2008**, *24*, 784–795, doi:10.1021/la702481v.
22. Pääkkö, M.; Ankerfors, M.; Kosonen, H.; Nykänen, A.; Ahola, S.; Österberg, M.; Ruokolainen, J.; Laine, J.; Larsson, P.T.; Ikkala, O.; et al. Enzymatic hydrolysis combined with mechanical shearing and high-pressure homogenization for nanoscale cellulose fibrils and strong gels. *Biomacromolecules* **2007**, *8*, 1934–1941, doi:10.1021/bm061215p.
23. Patel, M. Cumulative energy demand (CED) and cumulative CO2 emissions for products of the organic chemical industry. *Energy* **2003**, *28*, 721–740, doi:10.1016/S0360-5442(02)00166-4.
24. Håkan Stripple; Michelle Nerentorp; Wängberg, I. Environmental and Technical Evaluation with Life Cycle Assessment; IVL Swedish Environmental Research Institute: Gothenburg, Sweden, **2019**. https://www.praktikertjanst.se/globalassets/00.-praktikertjanst-koncernwebb/00.-bilder/life/nyheter/lca-life-cycle-assessment-hg-rid-life.pdf (accessed 21 July, 2021).
25. Life cycle inventories of chemicals. Ecoinvent version 2.0; 8; **2007**. http://www.ecoinvent.ch/ (accessed 21 April 2021).
